# Enhanced detection of lymphovascular invasion in small rectal neuroendocrine tumors using D2‐40 and Elastica van Gieson immunohistochemical analysis

**DOI:** 10.1002/cam4.935

**Published:** 2016-10-17

**Authors:** Yoshiyasu Kitagawa, Dai Ikebe, Taro Hara, Kazuki Kato, Teisuke Komatsu, Fukuo Kondo, Ryousaku Azemoto, Fumitake Komoda, Taketsugu Tanaka, Hirofumi Saito, Makiko Itami, Taketo Yamaguchi, Takuto Suzuki

**Affiliations:** ^1^ Endoscopy DivisionChiba Cancer CenterChibaJapan; ^2^ Division of Surgical PathologyChiba Cancer CenterChibaJapan; ^3^ Department of GastroenterologyFunabashi Central HospitalFunabashiJapan; ^4^ Pathology DivisionFunabashi Central HospitalFunabashiJapan; ^5^ Pathology DivisionSchool of MedicineTeikyo UniversityItabashiJapan; ^6^ Department of GastroenterologyKimitsu Chuo HospitalKimitsuJapan; ^7^ Department of GastroenterologyChiba Rosai HospitalIchiharaJapan; ^8^ Department of GastroenterologyChiba Kaihin Municipal HospitalChibaJapan; ^9^ Department of GastroenterologyChiba Cancer CenterChibaJapan

**Keywords:** D2‐40, Elastica van Gieson, lymphovascular invasion, rectal neuroendocrine tumors

## Abstract

Rectal neuroendocrine tumor (RNET) lymphovascular invasion (LVI) is regarded as an important predictor of nodal metastasis after endoscopic resection (ER). However, little is known about the frequency of immunohistochemical detection of LVI in RNETs. This study was performed to establish the actual detection of LVI rate in RNETs ≤10 mm and to evaluate associated clinical outcomes. We retrospectively reviewed the records for 98 consecutive patients treated by ER with a total of 102 RNETs ≤10 mm. Tissue sections were labeled with hematoxylin–eosin (HE) stain, the D2‐40 monoclonal antibody to evaluate lymphatic invasion, and Elastica van Gieson (EVG) stain to detect venous invasion. LVI detection rate by HE versus immunohistochemical analysis was compared. Follow‐up findings and clinical outcomes were also evaluated for 91 patients who were followed for ≥12 months. Lymphatic and venous invasion were detected using HE staining alone in 6.9% and 3.9% of patients, respectively, whereas they were detected using D2‐40 and EVG staining in 20.6% and 47.1% of the patients, respectively. Thus, the LVI detection frequency using D2‐40 and EVG staining (56.9%) was significantly higher than with HE (8.8%). Two out of seven patients who required additional surgery had regional lymph node metastases. However, among the 84 patients who were followed up without surgery, no distant metastases or recurrences were detected. Compared with HE staining, immunohistochemical analysis significantly increased the frequency of LVI detection in RNETs ≤10 mm. However, the clinical impact of LVIs detected using immunohistochemical analysis remains unclear. Clarification of the actual role of LVI using immunohistochemical analysis requires a patient long‐term follow‐up and outcomes.

## Introduction

Rectal neuroendocrine tumors (RNETs) have been reported more frequently in recent years, with greater RNET detection potentially stemming from the widespread use of screening endoscopy for colorectal cancer 1. RNETs demonstrate a broad range of clinical behavior, from benign and asymptomatic lesions to disseminated and highly metastatic cancers. Assessing tumor size 2, 3 and depth of invasion 4 is thought to be the simplest method for predicting future RNET, with those measuring ≤10 mm in diameter rarely metastasizing. Thus, RNETs ≤10 mm and confined to the submucosa (SM) are usually treated by endoscopic resection (ER). Following ER, lymphovascular invasion (LVI) is considered to be an important predictor of RNET nodal metastasis 5, 6, 7, 8. Previous studies have shown that RNETs ≤10 mm display very infrequent LVI 9, 10. Nonetheless, in a preliminary evaluation, we found that LVI was often detected by either hematoxylin–eosin (HE) staining or immunohistochemical analysis to detect specific lymphatic or venous endothelial markers. LVI in colorectal cancer is also considered to be an important predicator for lymph node metastasis; thus, immunohistochemical staining techniques are increasingly used to identify lymphatic channels and blood vessels due to the difficulty in recognizing lymphatic channels and veins using HE staining alone 11, 12, 13, 14. For example, immunostaining with the monoclonal antibody D2‐40 (D2‐40) can highlight the location of lymphatic endothelial cells and distinguish lymphatic channels from other small vessels. Similarly, venous walls are often identified using either Elastica van Gieson (EVG) or Victoria blue staining because of the resulting dark violet color taken on by the elastic fibers located in the venous wall with these techniques. Despite the importance of LVI as a prognostic factor after ER, there are few reports on the incidence of LVI with RNETs ≤10 mm as detected by immunohistochemical analysis 15. Likewise, limited information is available concerning patient outcomes following ER for RNETs in the absence of additional surgery. Thus, there are no published studies that evaluated potential correlations between LVI detected using immunohistochemistry and regional lymph node metastasis. The purpose of this study was to determine the frequency of LVI detection in RNETs ≤10 mm using D2‐40 and EVG staining and to evaluate clinical outcomes, including pathological results, with additional surgery following ER.

## Methods

### Patients

The study protocol conformed to the ethical guidelines of the Helsinki Declaration and was reviewed and approved by the local institutional review board. We retrospectively reviewed the records of 100 consecutive patients harboring 104 RNETs treated by endoscopic mucosal resection (EMR) or endoscopic submucosal dissection (ESD) between November 2005 and April 2015 at five hospitals in Japan. Patient data were included in the analyses if the RNETs were ≤10 mm in diameter. Specific patient demographic data extracted from the medical records included patient age, gender, endoscopic findings, and clinical outcomes.

### Tumor specimens

Tumor specimens resected by ER were routinely fixed with formalin, embedded in paraffin, cut into 2‐mm‐sections, and stained with HE. Histological evaluation of RNETs, including determination of tumor size, margin status, SM depth, and mitotic rate were all performed on HE‐stained tissue sections. The resection margin was evaluated according to TNM classification 16.

For this study, all archived tissue slides for RNETs ≤10 mm were reviewed and 1 cross‐section showing invasion into the deepest portion in the submucosal layer was selected. The corresponding paraffin blocks were cut into 4‐*μ*m‐thick serial sections that were subjected to immunohistochemical examination. The following monoclonal antibodies were used for immunohistochemistry: D2‐40 (dilution 1/40, BioLegend, San Diego, CA) and Ki‐67 (MIB‐1, Ready‐to‐Use, DAKO, Glostrup, Denmark). All staining was performed using an AutoStainer (EnVision System; DAKO). EVG staining was performed using a standard protocol.

Tumors were separated into two groups based on their World Health Organization 2010 classification 17: Grade 1 (≤2% on the Ki‐67 index) or grade 2 (3–20% on the Ki‐67 index). The presence of lymphatic channel invasion and venous invasion were initially determined based on the HE staining. After reviewing the HE staining, lymphatic invasion was re‐examined using D2‐40 staining and venous invasion was re‐examined using EVG staining.

All pathological slides were reviewed by two experienced gastrointestinal pathologists (D.I., M.I.) who were completely blinded to all clinical information pertaining to evidence of histologic LN metastases or distant metastases.

We evaluated samples for factors that are predictive of metastases, such as high mitotic index, LVI, and incomplete resection margin (R1) based on each patient's histologically confirmed diagnosis. In clinical practice, assessment of prognostic factors is mainly based on the evaluation by HE staining. Immunohistochemical evaluation has only been used for a subset of recent cases. As a general rule, additional surgery was recommended for patients with evidence that was predictive of metastases.

### Statistical analysis

**Figure 1:**
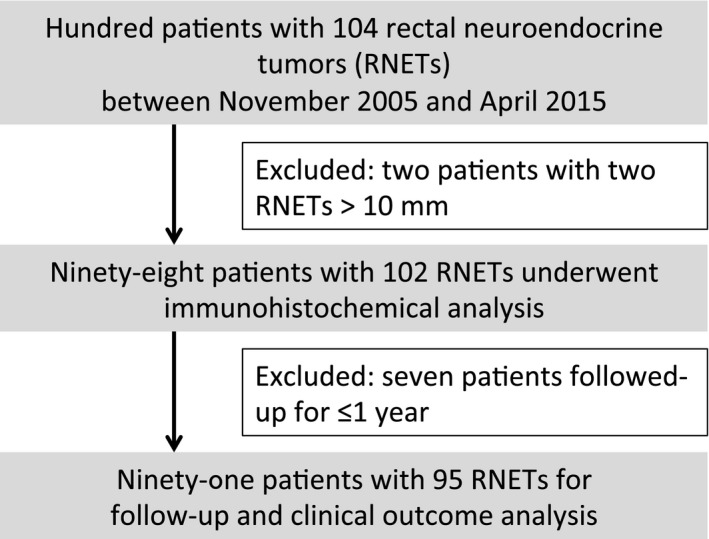
Flow diagram of included patients.

Analyses compared the frequency of LVI detection in RNETs ≤10 mm by immunohistochemical analysis using D2‐40 and EVG staining, with the detection frequency by HE staining. For the analysis of patient follow‐up results and clinical outcomes, we excluded seven patients with <12 months of follow‐up data, leaving 91 patients who were treated between 2005 and 2014 for subsequent analyses (Fig. 1). Clinical outcome data were collected from the electronic medical records of these patients. Incomplete and missing data were retrieved from referring physicians. Overall survival time was measured from the date of ER to the date of death or the last date of confirmed survival, and then the overall survival rate was calculated by Kaplan–Meier analysis. Significant differences between groups were evaluated using the Fisher's exact test. *P *< 0.05 for two‐tailed tests was regarded as significant.

## Results

### Clinicopathological characteristics

In total, 100 patients who underwent EMR or ESD were initially included in this study. Two patients had lesions >10 mm in diameter and were excluded from the analysis. Of the 98 patients with 102 RNETs ≤10 mm who were included in this study, 48 had undergone EMR and 54 had undergone ESD. All patients were asymptomatic and their lesions were incidentally identified during endoscopy. The median patient age was 61 years (range: 27–84) with a preponderance of males (*n *= 63, 64.3%). Of the 102 RNETs ≤10 mm, 11 (10.8%) were located in the upper rectum, and 91 (89.2%) were located in the lower rectum. Three lesions (2.9%) exhibited surface depression. The median tumor diameter was 5.0 mm (range: 1.0–10.0 mm). R0 resection was achieved for 73 lesions, Rx for 16 lesions, and R1 for 13 lesions. The median SM depth was 2000 *μ*m (range: 400–5500 *μ*m). In all the tumors**,** the mitotic count was very low, with grading based on tumor Ki‐67 index. Nine tumors demonstrated a Ki‐67 index of 3–4% with 93 Grade 1 RNETs and nine Grade 2 RNETs (Table 1).

**Table 1:** Clinicopathological features of patients

Age, median (range), years	61 (27–84)
Sex, *n* (%)	
Male	63 (64.3)
Female	35 (35.7)
Location, *n* (%)	
Upper rectum	11 (10.8)
Lower rectum	91 (89.2)
Depressed lesion, *n* (%)	
Present	3 (2.9)
Absent	99 (97.1)
Treatment, *n* (%)	
Endoscopic mucosal resection	48 (47.1)
Endoscopic submucosal dissection	54 (52.9)
Size, median (range), mm	5 (1–10)
Resection margin, *n* (%)	
R0	73 (71.6)
RX	16 (15.7)
R1	13 (12.7)
SM depth, median (range), *μ*m	2000 (400–5500)
WHO 2010 classification	
NET G1	93 (91.2)
NET G2	9 (8.8)

### Lymphatic invasion and venous invasion

**Figure 2:**
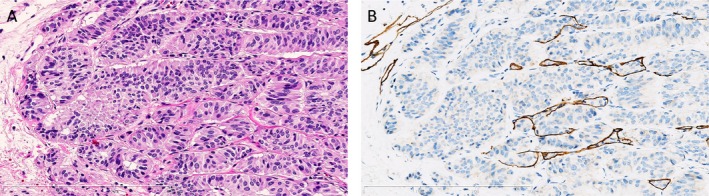
Identification of the involvement of lymphatic channel of rectal neuroendocrine tumor on serial tissue sections. (A) Identification of lymphatic invasion was difficult using only hematoxylin–eosin staining. Original magnification ×200. (B) D2‐40 immunohistochemical staining allowed for easier identification of lymphatic invasion by labeling the lymphatic epithelium. Original magnification ×200.

**Figure 3:**
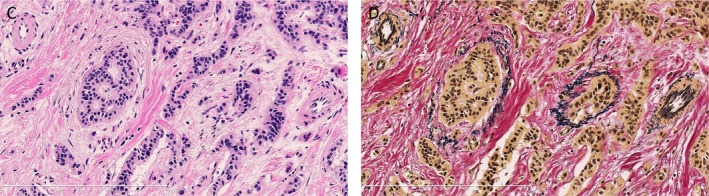
Identification of venous invasion of rectal neuroendocrine tumor on serial tissue sections. (C) Lymphatic invasion was difficult to identify using only hematoxylin–eosin staining. Original magnification: ×200. (D) Elastica van Gieson staining greatly improved the identification of venous invasion by dark staining of venous elastic fibers. Original magnification ×200. The labels for the figure panels should start with (A), then (B), independently for each figure.

The methods for highlighting lymphatic and venous invasion using D2‐40 and EVG staining in comparison with HE staining are presented in Figures 2 and 3, respectively. Using only HE staining, we detected lymphatic invasion in 6.9% (7/102) tumors and venous invasion in 3.9% (4/102) tumors. Using D2‐40 and EVG staining, lymphatic and venous invasion was detected in 20.6% (21/102) and 47.1% (48/102) of tumors, respectively. Finally, the LVI detection rate using D2‐40 and EVG staining was significantly increased compared with that using HE stain (56.9% vs. 8.8%, *P* < 0.0001, Table 2).

**Table 2:** Lymphatic and venous invasion detected by immunohistochemical staining using hematoxylin–eosin, D2‐40, and Elastica van Gieson

	Stain	Present (%)	*P* value
Lymphatic invasion	HE	7 (6.9)	0.0073
	D2‐40	21 (20.6)	
Venous invasion	HE	4 (3.9)	<0.0001
	EVG	48 (47.1)	
Lymphovascular invasion	HE	9 (8.8)	<0.0001
	D2‐40/EVG	58 (56.9)	

Data were analyzed using Fisher's exact test. HE, hematoxylin–eosin.

With regard to tumor size, the LVIs were detected significantly more frequently with tumors >5 mm than with those ≤5 mm (72.2% vs. 48.5%, *P* =0.0231, Table 3). The LVI detection rate was independent of WHO 2010 Classification (58.1% vs. 44.4%, *P* = 0.4944, Table 4).

**Table 3:** Frequency of lymphovascular invasion according to the tumor size

Size (mm)	*n*	Present (%)	*P* value
≤5	66	32 (48.5)	0.0231
>5	36	26 (72.2)	

Data were analyzed using Fisher's exact test. HE, hematoxylin–eosin.

**Table 4:** Frequency of lymphovascular invasion according to World Health Organization 2010 classification

WHO 2010 Classification	*n*	Present (%)	*P* value
NET G1	93	54 (58.1)	0.4944
NET G2	9	4 (44.4)	

Data were analyzed using the Fisher's exact test.

### Follow‐up and clinical outcomes

Among the 91 patients with 95 RNETs who underwent ER between 2005 and 2014, in whom follow‐up and clinical outcomes were analyzed, the median observation period was 51.7 months (range: 12–191 months). Among the 91 patients, 74 patients were evaluated using HE staining and 17 patients were evaluated using immunohistochemical analysis in routine clinical practice. Among the 74 patients with HE‐stained samples, 12 patients displayed other factors that were predictive of metastases. The major reason was an incomplete resection margin (*n *= 12). Among the 12 patients with prognostic factors, three underwent additional surgery and one was found to have regional lymph node metastasis (Table 5). Among the 17 patients who had immunohistochemical analysis, seven patients had factors that were predictive of metastases, with LVI as the most frequent factor (*n *= 7). Of the seven patients with prognostic factors, four underwent additional surgery and one was found to have regional lymph node metastasis (Table 6). The two metastatic tumors displayed LVI by immunohistochemical analysis. The other 12/19 patients with prognostic factors did not undergo surgery because of comorbidities (*n *= 2), refusal to undergo surgery (*n *= 6), or advanced age (*n *= 4).

**Table 5:** Pathological evaluation of additional surgical resection specimens using hematoxylin–eosin staining

Age, years	Sex	Size, mm	Resection margin	NET G1/G2	LVI (HE)	LVI (IHC)	LN metastasis
61	M	2	R1	G2	−	−	−
74	M	7	R1	G1	−	−	−
67	F	5	R1	G1	−	+	+

HE, hematoxylin–eosin; LVI, lymphovascular invasion.

**Table 6:** Pathological evaluation of additional surgical resection specimens using immunohistochemical analysis

Age, years	Sex	Size, mm	Resection margin	NET G1/G2	LVI (HE)	LVI (IHC)	LN metastasis
52	M	10	R0	G1	−	+	+
		7	R1	G1	−	−	
57	M	6	R0	G1	−	+	−
72	F	7	R0	G2	−	+	−
51	F	6	R0	G2	−	+	−

LVI, lymphovascular invasion.

**Figure 4:**
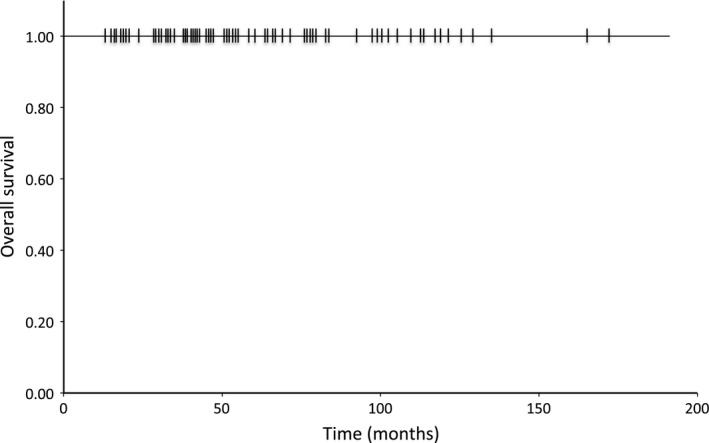
Kaplan–Meier overall survival curve for patients with rectal neuroendocrine tumors who underwent endoscopic resection.

Among the 91 patients, 84 patients were followed up without surgery. Surveillance colonoscopy was performed on 70 of the 84 patients (83.3%) after ER. No patients displayed local recurrence during the follow‐up period. For evaluation of metastatic disease, abdominal computed tomography (CT) and/or abdominal ultrasonography (US) were examined in 73 of the 84 patients (86.9%): 60 patients underwent abdominal CT and 22 patients underwent abdominal US. No patients demonstrated recurrence on abdominal CT and/or US during the follow‐up period. No patients died from RNETs or another cause during the study period and the 5‐year overall survival rate was 100% (Fig. 4).

## Discussion

This study established the LVI detection rate using immunohistochemical analysis of RNETs ≤10 mm using D2‐40 and EVG staining in addition to HE staining. Assessment of LVI in submucosal invasive colorectal cancer using D2‐40 and EVG staining was reported to double the detection rate compared to using HE staining alone 13. In this study, the detection rate of LVI using HE stain (8.8%) was similar to previously reported rate with RNETs ≤10 mm (0–8.1%) 9, 10. On the other hand, the LVI detection rate using D2‐40 and EVG staining (56.9%) was sixfold greater than with HE staining alone. Sekiguchi et al. 15 reported the frequent presence of LVI by immunohistochemical analysis in RNETs ≤13 mm (46.7%). The results of this study confirm the high prevalence of LVIs, even in small RNETs.

One of the reasons for the gap in LVI detection between HE staining and D2‐40 plus EVG staining may be that RNETs predominantly show a trabecular growth pattern and that areas of tumor cell lymphatic invasion may be difficult to identify using HE staining alone (Fig. 2). Similarly, HE staining does not specifically label the elastic fibers present in the venous wall, resulting in difficulty with identifying venous invasion (Fig. 3). Because of this unique pathological character of RNETs and the limitations of HE staining, additional immunohistochemical assessments are required to accurately identify LVIs in RNETs ≤10 mm. While our study demonstrated that the LVI detection rate increased with increasing tumor size, LVIs were still detected immunohistochemically in approximately 50% of RNETs ≤5 mm. Thus, we speculate that RNETs may potentially harbor LVIs even at their earliest stages and that previous reports may have underestimated the actual frequency of LVIs because of the use of HE staining. We believe that our immunohistochemical analysis results provide a more accurate estimate of LVI frequency in RNETs ≤10 mm.

In general, tumor size and depth of invasion are regarded as factors that are significantly predictive of malignancy in localized RNETs 2, 3, 4. According to previous studies, the risk of metastasis was 2–3% for tumors ≤10 mm, 10–15% for tumors of 11–20 mm, and 60–80% for tumors >20 mm 2, 18. Also, a survival analysis including the nearly 5000 cases in the SEER database found a 97% 5‐year survival rate among patients with tumors ≤20 mm that were confined to SM 19. Based on these studies, RNETs ≤10 mm that have no preinterventional lymph node invasion are good candidates for ER and no further treatment is recommended if R0 resection is achieved 6, 20. A recent multivariate analysis showed that, in addition to the tumor size and depth of invasion, LVI is an important predictor for lymph node metastasis 5. Indeed, the updated European Neuroendocrine Tumor Society (ENETS) consensus guidelines and the Japanese Neuroendocrine Tumor Society guidelines (JNETS) recommend additional radical surgery combined with lymph node dissection if LVI was detected, even for RNETs ≤10 mm 7, 8. However, these guidelines may be based on data from assessments based on HE staining. If we apply these guidelines to our cases, more than 50% of patients would require additional radical surgery because of the presence of LVI after ER. Sekiguchi et al. 15 reported that 90 RNETs treated by ER were followed up without additional surgery, and no metastasis or recurrence was detected during a median follow‐up period of 67.5 months despite the presence of LVI in nearly half of the lesions. In this study, 84 patients were followed up without surgery and there were no instances of cause‐specific death or recurrence of malignancy over a median follow‐up period of 51.7 months. These results showed an excellent prognosis following ER for patients with RNETs. Rectal surgery is more invasive than colonic surgery. Since the introduction of abdominoperineal resection by Miles in the 1920s, this procedure has been the standard treatment for permanent stomas 21. More recently, advanced anus preserving, low anterior resection, and intersphincteric resection have become more common with colostomies more frequently avoided 22, 23. Nevertheless, previous reports have shown that some patients experience disordered defecation after low anterior resection or intersphincteric resection 24, 25. Increasing standardization has been recommended in relation to surgery for colorectal cancer 26. Considering the risk for complications, the role of additional radical surgery for RNETs ≤10 mm with LVI detected using immunohistochemical analysis requires broader discussion.

On the other hand, our study demonstrated that two of the seven patients who underwent additional surgery had regional lymph node metastasis, and that both metastatic tumors harbored LVI using immunohistochemical analysis. There have been a few reported cases showing that RNETs ≤10 mm that are confined to SM may already have node‐positive disease and may develop recurrence or metastasis after a long latency period 27, 28. From these findings, we cannot exclude the risk of nodal metachronous metastasis, even in patients with small RNETs. Thus, we emphasize that, following ER, patients should adhere to follow‐up surveillance, even with the removal of small tumors.

The important limitations of this study are its retrospective design and incorporation of a follow‐up period that was insufficient to evaluate the RNET clinical outcomes, considering the usual indolent clinical course. Thus, we cannot exclude the presence of clinically undetectable, minute metastatic lesions in the patients treated without surgery. Thus, the clinical impact of LVIs detected using immunohistochemical analysis remains unclear. Further studies involving a larger number of patients and extended follow‐up periods would be required to evaluate the importance of immunohistochemical analysis in determining the exact risk for metastasis in patients with RNETs ≤10 mm.

In conclusion, compared with HE staining, D2‐40 and EVG staining significantly increase the LVI detection rate in RNETs ≤10 mm. Additional prospective studies are required to clarify the role of LVIs detected using D2‐40 and EVG.

## Conflict of Interest

The authors made no disclosures.
